# New advances on Au–magnetic organic hybrid core–shells in MRI, CT imaging, and drug delivery

**DOI:** 10.1039/d1ra00415h

**Published:** 2021-02-05

**Authors:** Fatemeh Mohajer, Ghodsi Mohammadi Ziarani, Alireza Badiei

**Affiliations:** Department of Physics and Chemistry, Faculty of Science, University of Alzahra Tehran Iran gmohammadi@alzahra.ac.ir f.mohajer@alzahra.ac.ir +98 21 8041575; School of Chemistry, College of Science, University of Tehran Tehran Iran

## Abstract

Magnetic nanoparticles have been widely studied for various scientific and technological applications such as magnetic storage media, contrast agents for magnetic resonance imaging (MRI), biolabelling, separation of biomolecules, and magnetic-targeted drug delivery. A new strategy on Au–magnetic nano-hybrid core–shells was applied in MRI, CT imaging, and drug delivery, which has been received much attention nowadays. Herein, the designing of different magnetic core–shells with Au in MRI and cancer treatment is studied.

## Abstract 2

Magnetic nanoparticles have been studied for scientific and technological applications such as magnetic storage media, contrast agents for magnetic resonance imaging, biolabelling, separation of biomolecules, and magnetic-targeted drug delivery.

## Graphical abstract

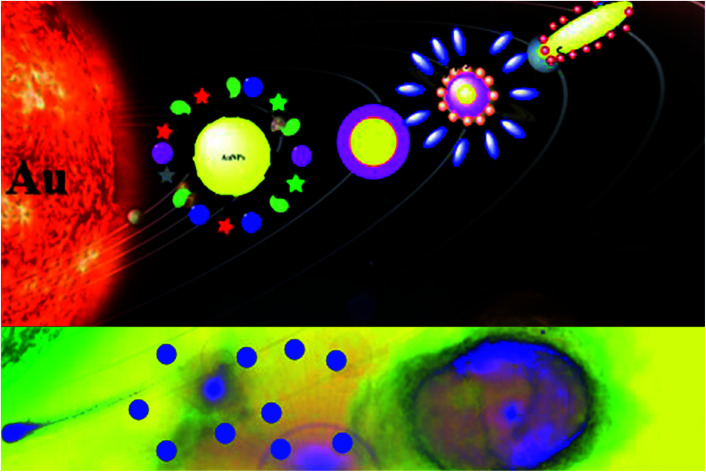

## 1. Introduction

There are various nanoparticles for medical applications, including carbon dots^1,2^ and organic core/shell nanoparticles. Among all these materials, Fe_3_O_4_ (ref. 3) and MnFe_2_O_4_ (ref. 4) gain the first step in terms of usage. However, TiO_2_,^5^ SnO_2_,^6^ Ag,^7^ Au,^8^ S,^9^ BaTiO_3_,^10^ and ZnO^11^ are known for their electrical applications. Through the coating process, various materials such as polyaniline (PA),^12^ polystyrene (PS), graphene,^6^ oleic acid, polyvinylpyrrolidone (PVP), hyperbranched aromatic polyamide (HBP),^10^ poly(methylmethacrylate) (PMMA), are differently used, and the provided materials are used extensively for batteries, optical sensors, and magnetic imaging. The metal–organic frameworks (MOFs) have received much attention in terms of bioimaging in living cells, as porous functional materials, which have large surface areas, high porosity, fluorescence functionalities, and good biocompatibility. Organic or inorganic fluorescent materials such as fluorescent dyes, quantum dots, and metal nanoclusters can also be applied in medicine for bioimaging.

**Fig. 1:**
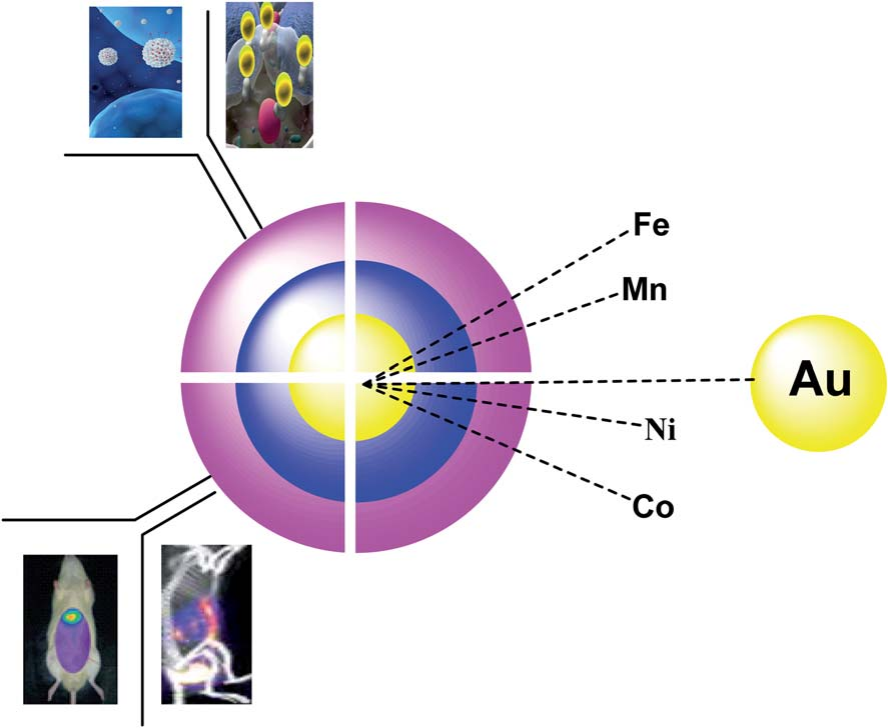
The application of the different nanoparticles as core–shells.

The importance of magnetite nanoparticles (Fe_3_O_4_ NP_S_) was extensively studied due to their many biomedical uses in cancer cells, magnetic resonance imaging (MRI) as a contrast agent, drug delivery, and hyperthermia treatment. In terms of toxicity and susceptibility, magnetite NPs are safe and have superparamagnetic applications.^13–16^ For improving the stability and dispersing the NPs in water, Fe_3_O_4_ NP was coated with Au NPs to improve the stability and dispersion of the NPs in aqueous media as efficient materials due to their biocompatibility, stability, resistance, which provided the potential medical requests.^17–20^ Gold nanoparticles (AuNPs) have received much attention because of their unique physicochemical activities such as biological, chemical, and biomedical implementations. AuNPs have been used for sensing, catalysis, imaging and diagnosis, and therapy (Fig. 1).^21^

Core–shell (CS) nanostructures have received much attention due to the known constituents and configuration.^22^ In this area, the immunization of Au on Fe_3_O_4_@ promoted the optical properties of computed tomography (CT) and MRI, which increased the resolution in 3D visual images in this process.^23,24^ Through the importance of bioimaging in living cells, metal–organic frameworks (MOFs) were used which organic or inorganic fluorescent compounds like quantum dots, fluorescent dyes, nanosheets, and metal nanoclusters can provide modified MOFs to give fluorescent nano-composites for an imaging agent.^25^ In continuing our previous work,^26,55–59^ we decided to introduce another application of core shells, including Au–magnetic organic hybrid in MRI, CT imaging, and drug delivery.

## 2. Au–magnetic organic hybrid core–shells

### 2.1. Fe_3_O_4_@SiO_2_@Au NPs

**Scheme 1:**
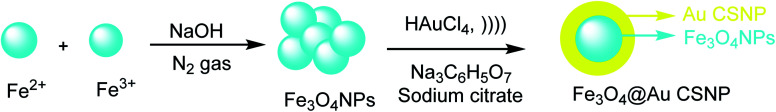
The preparation of Fe_3_O_4_@Au by sonochemical method.

Fe_3_O_4_ NP was provided by ferrous (Fe^2+^) and ferric (Fe^3+^) through the co-precipitation method^27^ with sodium hydroxide (NaOH) under inert nitrogen gas at room temperature. To obtain the Fe_3_O_4_ @Au CSNPs, the Fe_3_O_4_ was added to the HAuCl_4_ solution in Na_3_C_6_H_5_O_7_ and sodium citrate (CS) under sonicated conditions. According to the result, Fe_3_O_4_@Au CSNPs are suitable for CT and MRI imaging. The importance of this method is to give Fe_3_O_4_@Au CSNPs through the rapid sonochemical synthesis (Scheme 1).^28^

### 2.2. Fe_3_O_4_@SiO_2_@Au NPs

**Scheme 2:**
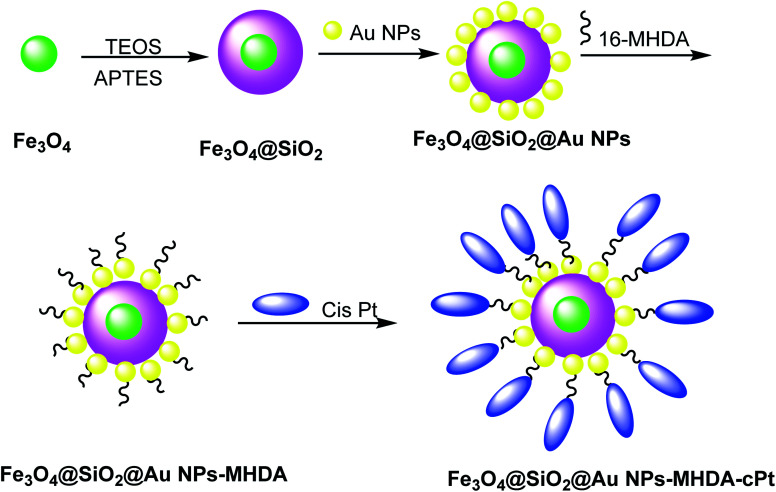
The preparation of Fe_3_O_4_@SiO_2_@Au NPs–MHDA–cPt.

Cisplatin (cPt) was doped on Fe_3_O_4_@SiO_2_@Au to cure cancer, which was detected through MRI images. Hydrophobic Fe_3_O_4_ NPs were synthesized by a thermal decomposition method^29,30^ using FeCl_3_·6H_2_O and sodium oleate in ethanol/hexane. In the next step, Fe_3_O_4_@SiO_2_ was obtained through the reverse microemulsion method.^31^ In this method, the mixture of Igepal CO-520 in cyclohexane and ammonium hydroxide was added to Fe_3_O_4_ NPs, followed by the addition of tetraethoxysilane (TEOS) and (3-aminopropyl)triethoxysilane (APTES) to yield Fe_3_O_4_@SiO_2_ core–shell nanostructures, which were added to Au NPs^32^ to obtain Fe_3_O_4_@SiO_2_@Au NPs. In the next step, 16-mercaptohexadecanoic acid (16-MHDA) as a linker with 16 carbon atoms containing thiol and carboxyl group was linked to the Au NPs surface and cPt, respectively (Scheme 2).^33^ The functionalized Fe_3_O_4_@SiO_2_@Au NPs are assessed in photothermal cancer therapy by the irradiation of two colon cancer cell lines (SW480 and SW620) with a laser (*λ* = 808 nm, *W* = 100 mW cm^−2^). It is found that laser irradiation showed the mortality of cancer cells. Because of the synergic effect of cPt and Au NPs and laser irradiation, the functionalized Fe_3_O_4_@SiO_2_@Au NPs are used for potential (MRI)-guided stimulated chemo-photothermal treatment of cancer.

### 2.3. Core–shell iron–gold (Fe@Au)

**Scheme 3:**
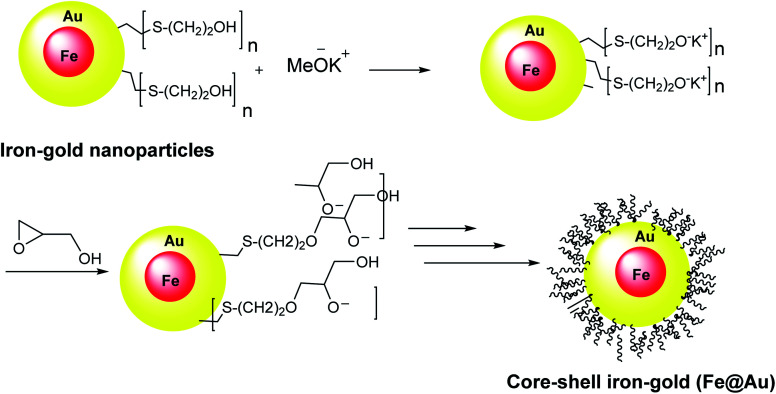
The preparation of core–shell iron–gold (Fe@Au).

Core–shell iron–gold (Fe@Au) structures were used for MRI imaging and targeted drug delivery. They were obtained through the reverse micelle method. In this method, the iron–gold nanoparticles were coated with polyglycerol, thiol, and polymerized glycidol. Spherical core particles of iron with a thin layer of gold shell were decorated with 2-mercaptoethanol, which was linked to Au from S head to yield the Au–S on the core–shell surfaces (Scheme 3).^34^

### 2.4. Fe_3_O_4_@SiO_2_PrNH_2_@Au

**Scheme 4:**
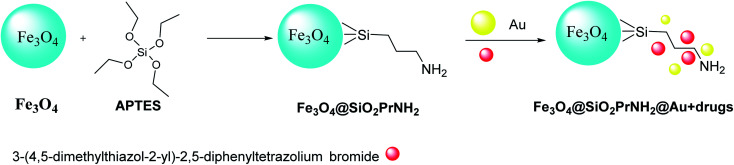
The preparation of Fe_3_O_4_@SiO_2_PrNH_2_@Au + drugs.

**Scheme 5:**
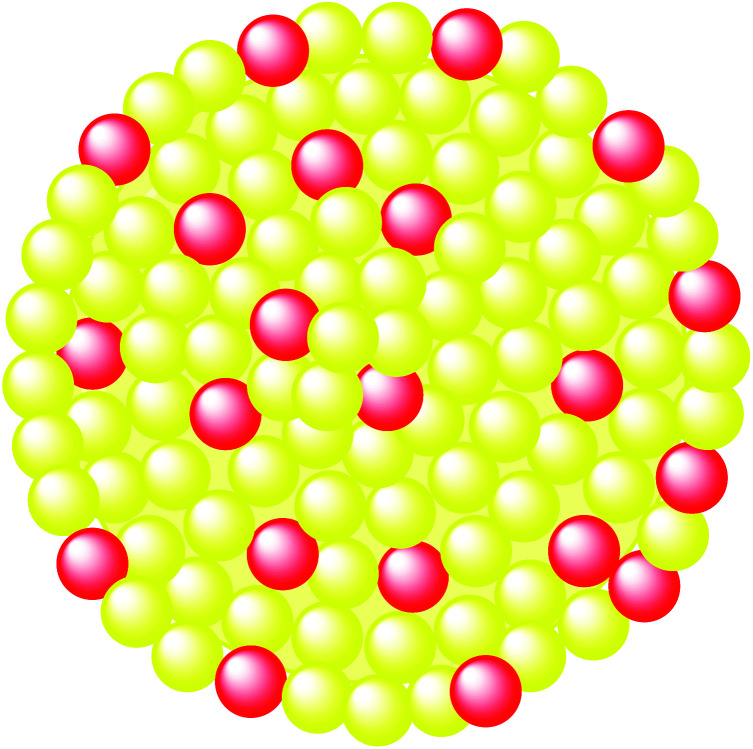
The structure of designed Fe_3_O_4_@Au.

Keshtkar *et al.*^37^ designed Fe_3_O_4_@Au by magnetic iron oxide nanoparticles through the co-precipitation process,^35^ followed by silica according to the Stöber method.^36^ Consequently, Fe_3_O_4_ nanoparticles were functionalized by (3-aminopropyl)triethoxysilane (APTES) to provide Fe_3_O_4_@SiO_2_PrNH_2_, which was added to the suspension of gold nanoparticles in H_2_O to produce Fe_3_O_4_@SiO_2_PrNH_2_@Au. Then, 3-(4,5-dimethylthiazol-2-yl)-2,5-diphenyltetrazolium bromide as human hepatocellular carcinoma was immobilized on the surface of Fe_3_O_4_@SiO_2_PrNH_2_@Au to give Fe_3_O_4_@SiO_2_PrNH_2_@Au + drugs, which was applied as MRI and CT agent (Scheme 4). Fe_3_O_4_@SiO_2_PrNH_2_@Au was provided through the synthetic strategy according to the laser ablation in liquid (LAL) as a green route to achieve NPs in one step (Scheme 5).^38–41^

### 2.5. Fe/Fe_3_O_4_PrNH_2_@Au

**Scheme 6:**
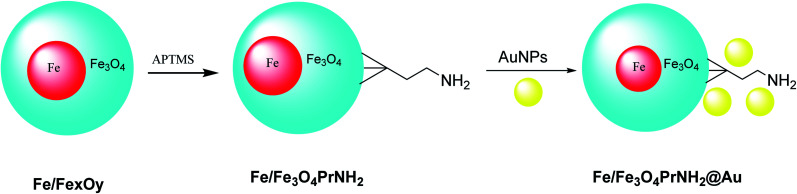
The preparation Fe/Fe_3_O_4_PrNH_2_@Au nanoparticles.

Iron/iron oxide nanoparticles Fe/Fe_*x*_O_*y*_@Au were provided through the electric arc discharge method. Then, particles were mixed with (3-aminopropyl)trimethoxysilane (APTMS) under sonicated conditions to provide Fe/Fe_3_O_4_PrNH_2_, which was functionalized by gold nanoparticles^42^ to provide Fe/Fe_3_O_4_PrNH_2_@Au. The designed Fe/Fe_3_O_4_PrNH_2_@Au nanoparticles as bifunctional magnetic plasmonic nanostructures were used in magnetic resonance imaging and magneto-optical thermal therapies (Scheme 6).^43^

### 2.6. Au@Bi_2_S_3_ NBs

**Scheme 7:**
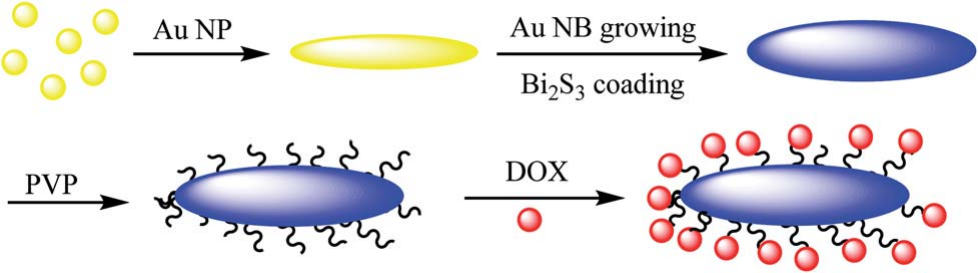
The preparation of NR Au@Bi_2_S_3_ NBs.

Au@Bi_2_S_3_–PVP nano bone, which can be used as a multimodal imaging agent for PT/PA/CT image, was reported by Ouyang *et al.*^45^ In the first step, Au NPs were synthesized^44^ and mixed with l-ascorbic acid (AA), hexamethylenetetramine, and thioacetamide (TAA) to give Au NR Au@Bi_2_S_3_ NBs as the theranostic agent in cancer therapy, which was added to bismuth acetate to give Au@Bi_2_S_3_ core–shell NBs, followed by mixing with PVP to produce Au@Bi_2_S_3_–PVP NBs nano bone as an imaging agent for applying in the tumor cells (Scheme 7). The structure can be used as a nanocarrier for anticancer drugs (DOX) to be released at a special pH.

### 2.7. d-Au@Gd&RGD

**Scheme 8:**
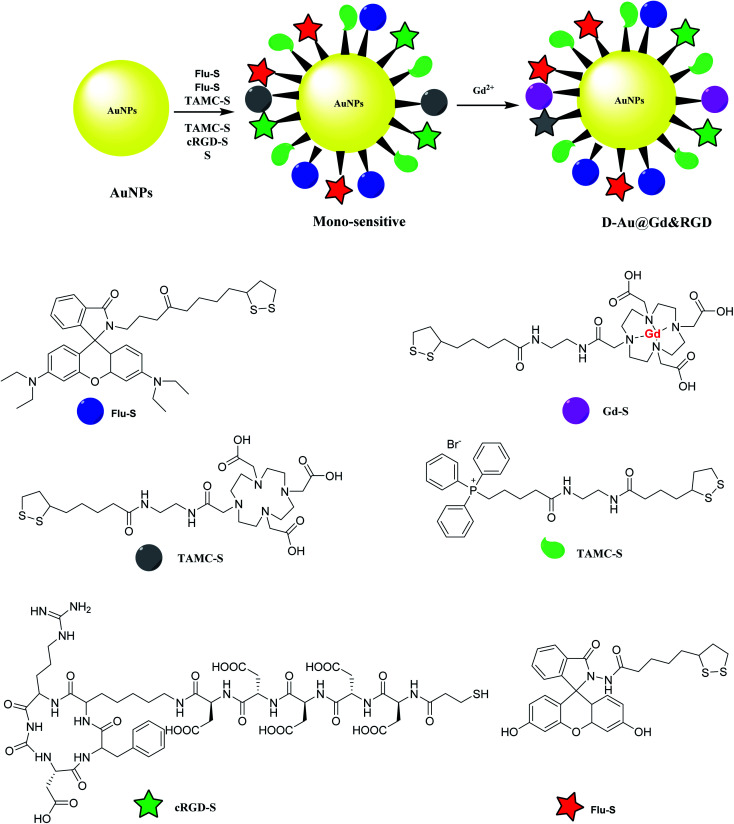
The preparation of d-Au@Gd&RGD.

Gold nanoparticles was provided to be functionalized by mitochondria-targeting group (Mito-S), rhodamine B derivative (RH-S), fluorescein derivative (Flu-S), tetraaza macrocyclic compounds (TAMC-S), cyclic arginine–glycine–aspartic acid peptide (cRGD-S) to yield a mono-sensitive compound. The latter was treated with Gd^2+^ to provide d-Au@Gd&RGD as the dual-sensitive structure. The application of the synthesized compound was used in MR and fluorescence imaging for a tumor in mice for tumor imaging and penetrate the blood–brain barrier (BBB) for central nervous system (CNS) problems (Scheme 8).^46^

### 2.8. APG@OVA NPs

**Scheme 9:**
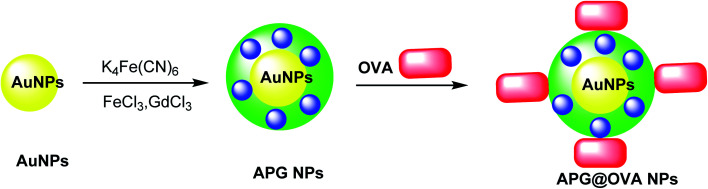
The preparation of APG@OVA NPs.

The multifunctional gadolinium-doped Au@Prussian blue nanoparticles (Au@PB–Gd@OVA) were designed as MR/SERS bimodal agents. Prussian blue nanoparticles (PB) as cyanide (CN)-bridged coordination polymer were immobilized onto the AuNP core to give a background-free surface-enhanced Raman scattering (SERS) signal. The presence of doped Gd^3+^ provided a sensitive agent for MRI. Through the coating of ovalbumin (OVA) physically, APG@OVA NPs were provided.^47^ First step: The Au NPs were prepared through the classic sodium citrate reduction process using sodium citrate and HAuCl_4_ solutions. Second step: K_4_[Fe(CN)_6_] and FeCl_3_ were applied as the Prussian blue nanoparticles (PB) agent, which was reacted with GdCl_3_. These two solutions were mixed to provide APG NPs, which was added to the OVA solution to yield Au@Prussian blue-Gd@ovalbumin nanoparticles (APG@OVA NPs) (Scheme 9).^48^

### 2.9. Au@MnO_2_

**Scheme 10:**
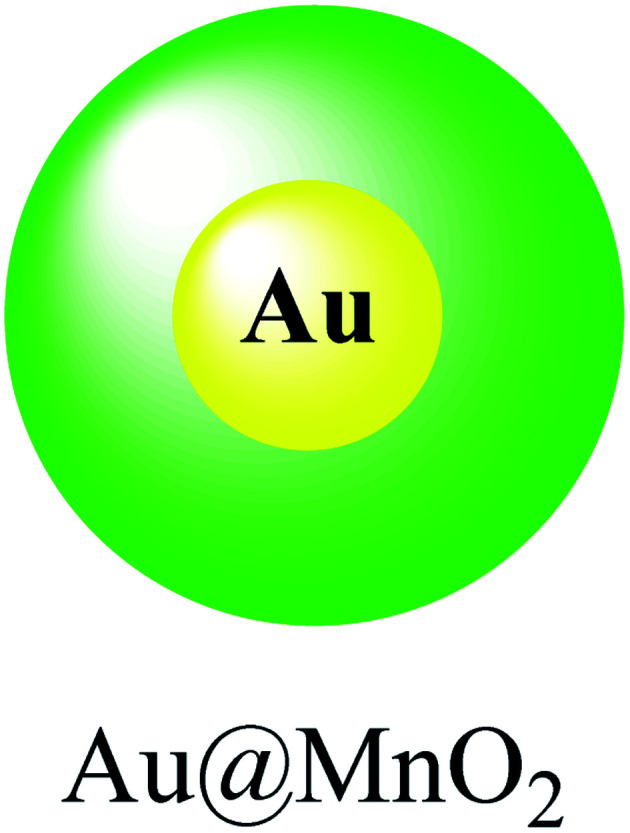
The preparation of Au@MnO_2_.

In this study, a gold@manganese dioxide (Au@MnO_2_) as core–shell structure was synthesized, which was functionalized by glutathione GSH as a theranostic agent in photoacoustic and magnetic resonance study. The GSH-triggered Au@MnO_2_ was applied in photoacoustic and MRI as a smart theranostic nanostructure for cancer diagnosis and treatment (Scheme 10).^49^

**Scheme 11:**
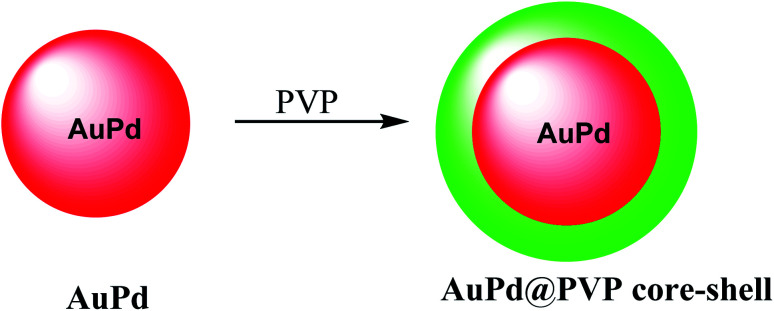
The preparation of AuPd@PVP core–shell nanoparticles.

The nanostructure of AuPd@PVP is important due to their similar magnitudes to the biomolecules, which was used for in biotechnology and medicine. The chloroauric acid hydrated solution was added to the Au precursor; and then disodium tetrachloropalladate was mixed with ascorbic acid as a reducing agent to give AuPd (Scheme 11).^50^ The resulting product AuPd was mixed with polyvinyl pyrrolidone (PVP) as a surfactant to give AuPd@PVP nanoparticles for biocompatibility *in vivo* and *in vitro* study in photothermal therapy (PTT) and radiotherapy (RT). Therefore, PTT and RT could be applied for cancer therapy. The AuPd@PVP NPs have photothermal therapy (PTT) activity under NIR laser irradiation at a low power. Moreover, the NPs could be applied in radiotherapy (RT) as the sensitizer agent. Through PTT and RT, AuPd@PVP core–shell nanoparticles could be efficient for cancer therapy.

### 2.10. Fe_3_O_4_@Au

**Scheme 12:**
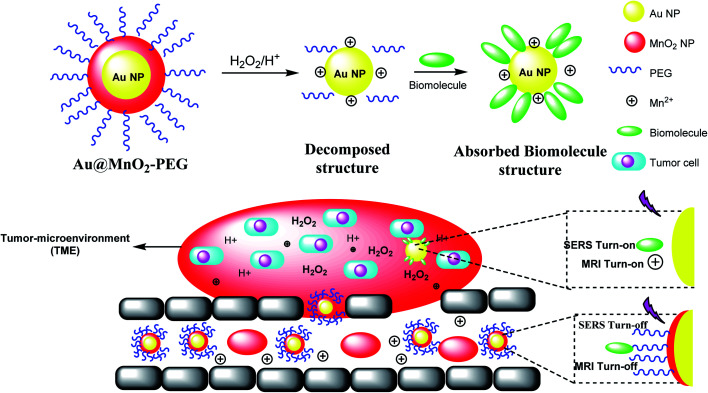
The preparation of SR-SERS nanoprobe.

The manganese dioxide-encapsulated gold nanoparticle (Au@MnO_2_ NP) was decorated by polyethylene glycol (PEG) to provide Au@MnO_2_–PEG and decomposed into the naked Au NPs and Mn^2+^ in acidic media. The resulting structure was absorbed by biomolecules to give a stimuli-responsive surface-enhanced Raman scattering (SR-SERS) nanoprobe (Scheme 12). The SR-SERS probes determined the difference between tumor and normal tissues by accuracy and even in different growth steps.

A stimulus-responsive surface-enhanced Raman scattering (SR-SERS) nanoprobe was designed to diagnose the tumor cells. The SR-SERS probes were activated by the tumor microenvironment (TME); however, in other tissues, which were highly selective to tumors, there was no activation. As a result, TME-triggered exposure with Au NPs and biomolecules could meaningfully increase; moreover, the Raman fingerprints show the complete information, compared with the traditional molecular method. The importance of the SR-SERS probes is to make a difference between tumors and healthy tissues, which might be applicable for the treatment of cancers (Scheme 12).^46^ In fact, Au@MnO_2_ nanoparticles were produced through a layer-by-layer approach. Thus, the obtained Au@MnO_2_-PEG nanoparticles treated with H_2_O_2_ in the tumor to provide Mn^2+^ for improving *T*_1_-weighted MR imaging to create O_2_ for removing the cancer cells to X-rays. Therefore, Au nanoparticles increase the X-ray energy in tumor cells, and MnO_2_ reacts with endogenous tumor H_2_O_2_ to create O_2_ in hypoxia-associated RT resistance. Through the combination of gold nanoparticles and O_2_ generation by MnO_2_ shells, Au@MnO_2_-PEG core–shell nanoparticles, there is a good tumor therapeutic effect.

### 2.11. EGaIn–Au NPs

**Scheme 13:**
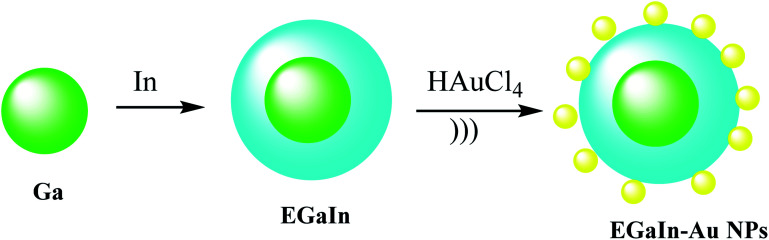
The preparation of EGaIn–Au NPs.

To give nano-composites through liquid metals (LM), a mixture of gallium indium–Au nanostructures (EGaIn–Au NPs) was used for providing radio-photothermal cancer treatment. In this process, Au nanodots were reduced onto the mixture of gallium indium (EGaIn) NPs surface to achieve EGaIn–Au NPs. This strategy might open a new door to a LM-based nano-composite. The EGaIn NPs were designed through the probe-sonicating method in xanthan gum solution after centrifuging the spherical EGaIn NPs, and HAuCl_4_ solution was mixed with EGaIn NPs for growth of Au NPs by the interfacial galvanic replacement reactions to give EGaIn–Au nano-composites (Scheme 13).^51^ It was proved that the EGaIn–Au nano-composite was used to respond the X-ray and NIR (near-infrared laser) irradiation. The nano-composite with photothermal conversion and radiosensitization ability destroy cancer cells to cure. However, healthy tissues are damaged, they used for decreasing the growth of tumor tissues by NIR, and X-ray treatment in photothermal therapy and radiotherapy.

### 2.12. I–Pd@Au–PEG–FA nanosheets

**Scheme 14:**
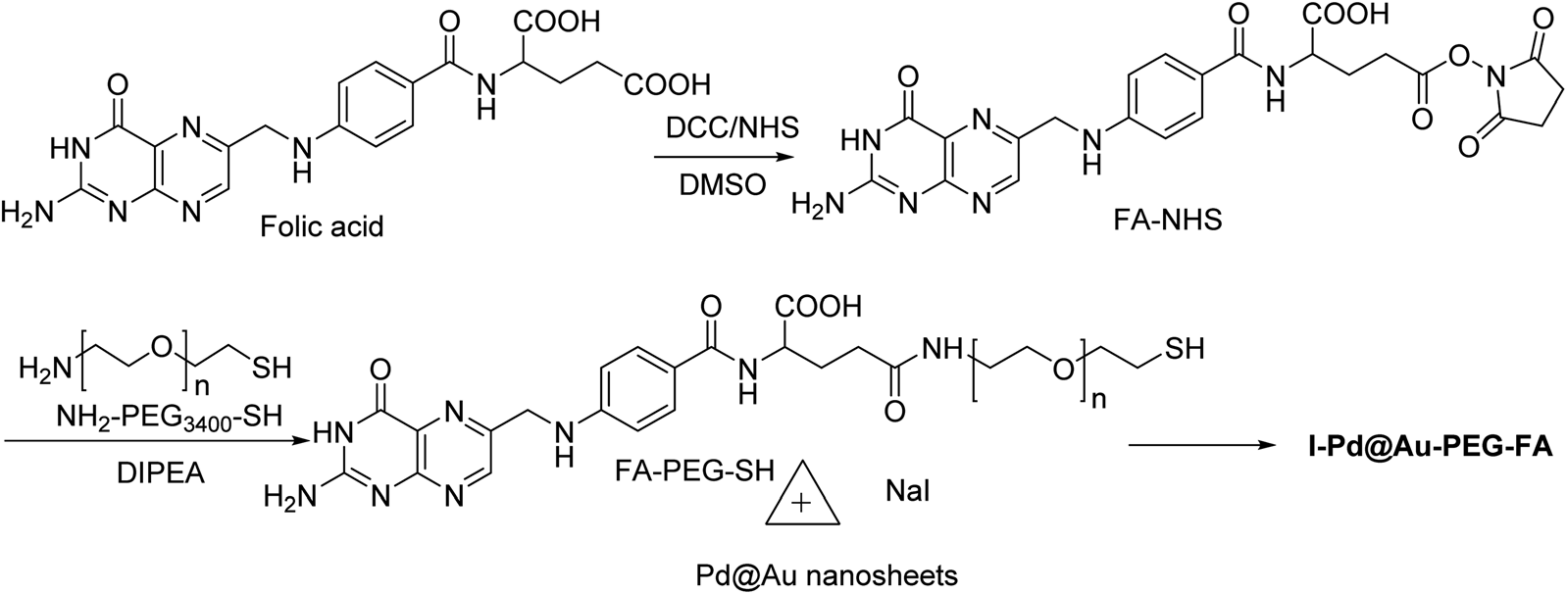
The preparation of core–shell I–Pd@Au–PEG–FA.

**Scheme 15:**
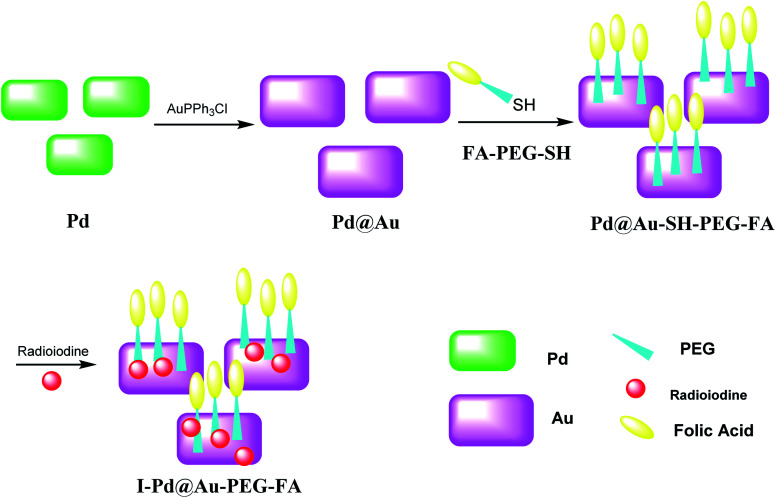
The preparation of I–Pd@Au–PEG–FA nanosheets.

Pd@Au–PEG–FA nanosheets were used as CT image contrast agents. The Pd@Au nanosheets were synthesized according to previous reports.^52–54^ FA–NHS was obtained from folic acid (FA), *N*-hydroxysuccinimide (NHS), and dicyclohexylcarbodiimide (DCC) in DMSO in the dark to provide FA–NHS, which was added to the NH_2_–PEG–SH to provide Pd@Au–PEG–FA nanosheets, and then it was mixed with FA–PEG–SH to provide Pd@Au–PEG–FA nanosheets. Preparation of I–Pd@Au–PEG–FA nanosheets was accomplished through the reaction of Pd@Au–PEG–FA with radioiodine at room temperature, which was used to load radioiodine by ^125^Na^131^I at room temperature (Schemes 14 and 15).^51^ I–Pd@Au–PEG–FA nanosheets were then applied on detecting the plaques by reforming the 2D multifunctional structure by FA on the surface and evaluate the target specificity for the activated macrophages; the targeted probes show that 2D Pd@Au nanosheets have superior pharmacokinetic to achieve the cure effect.

## 3. Conclusion

Au nanoparticles received much attention for cancer treatment and MRI due to their high chemical stability, biocompatibility, and affinity for binding with thiol terminal groups of various organic compounds. Moreover, these mixtures give the magnetic and plasmonic properties to nanoparticles for diagnostics and therapeutic applications. However, the currently available synthesis methods for these nanoparticles are based on organic compounds.

## Conflicts of interest

The authors declare that there is no declaration of competing interest in this paper.

## Supplementary Material
